# Improving the Assessment of Childhood Adversity: Factors Influencing Questionnaire and Interview Method Concordance

**DOI:** 10.1007/s40653-026-00817-2

**Published:** 2026-03-04

**Authors:** Austen McGuire, George M. Slavich, Neal Kingston, Damion Grasso, Yo Jackson

**Affiliations:** 1 National Crime Victims Research and Treatment Center, Department of Psychiatry & Behavioral Sciences, Medical University of South Carolina, Charleston, SC 29425 USA; 2 Department of Psychiatry and Biobehavioral Sciences, University of California, Los Angeles, 760 Westwood Plaza, Los Angeles, CA 90095 USA; 3 Department of Educational Psychology, University of Kansas, 1122 West Campus Rd, Lawrence, KS 66045 USA; 4 Achievement and Assessment Institute, University of Kansas, 1515 St. Andrews Drive, Lawrence, KS 66047 USA; 5 Department of Psychiatry, University of Connecticut School of Medicine, 263 Farmington Avenue, Farmington, CT 06030 USA; 6 Department of Psychology, The Pennsylvania State University, University Park, PA 16802 USA

**Keywords:** Childhood adversity, Trauma, Stress, Children, Assessment, Methodology

## Abstract

Although caregiver reports of adverse life events (ALEs) in young children yield notable discrepancies for face-to-face interview vs. paper-and-pencil questionnaire assessments, the factors contributing to these differences are not well understood. The present study addressed this knowledge gap by examining multiple factor domains for caregivers (e.g., caregivers’ assessment beliefs, mental health, demographics) that might play a role in these discrepancies. Participants were 57 caregivers (*M*_age_ = 33.72; 96.5% biological mothers; 61.4% Black/African American) of pre-school and school-age children who completed an interview and questionnaire ALE assessment, as well as measures of mental health challenges and research participation beliefs (e.g., positive experience, privacy, research rights). Concordance between formats at the participant level was mostly in the moderate range. Results suggested that participants were more likely to believe they could exercise their research rights (e.g., know they could terminate the assessment at any time) during the interview format relative to questionnaire format. Caregiver mental health, race-congruence with the interviewer, and participation beliefs were not significantly associated with total agreement. However, participants from lower federal poverty levels tended to demonstrate lower agreement between formats, compared to participants from higher federal poverty levels. Taken together, these findings highlight the need for dual approaches to assess ALEs in children when relying on caregiver report, as well as to ensure the aspects of the ALE administration procedure (including before and after the ALE assessment) are similar across assessment formats. This may be especially important when working with families from low-income backgrounds to improve detection of ALEs in young children.

**Supplementary Information:**

The online version contains supplementary material available at 10.1007/s40653-026-00817-2.

Accurate assessment of adverse life events (ALEs; e.g., maltreatment, violence exposure, and death of loved ones) is critical to identifying exposed children, understanding why children experience maladjustment following ALEs, and connecting these children to services. For young children (i.e., approximately < 8 y.o.), assessment often relies on caregivers because of these children’s developmental level. For example, young children may not remember ALEs that occurred to them in the distant or even recent past, or may not have the language skills to know what types of events are being assessed for (e.g., Bartlett, 2020; Ogle et al., 2008). Given the importance of relying on caregiver report for the assessment of ALEs in young children, it is necessary to evaluate and understand the factors that may influence caregiver reporting behaviors.

An important consideration in this assessment context is understanding how differences in assessment format may influence a caregiver’s endorsement of ALEs (Oh et al., 2018). For caregivers reporting on young children’s ALEs, the two most utilized assessment modalities are (a) face-to-face interviews and (b) paper-and-pencil questionnaires (e.g., Eklund et al., 2018; Oh et al., 2018). There are several notable differences between interviews and questionnaires and each method has its proposed strengths and weaknesses, resulting in the widespread use of both formats. For example, interview approaches are thought to permit researchers greater opportunities to capture detailed information and build rapport through in-depth conversations on ALEs (e.g., Harkness & Monroe, 2016). However, this can come with important trade-offs, as interviews can be time consuming and require greater resources to implement and analyze in research. In contrast, paper-and-pencil questionnaires may be shorter and less burdensome to reporters and researchers compared to interviews, which can be important in research studies with limited budgets or when working with challenging to reach populations (e.g., youth in foster care; Milne & Collin-Vézina, 2015; Oh et al., 2018). Yet, the information obtained from questionnaires may be limited in scope because of their brevity. Unsurprisingly, several studies have documented significant differences in ALE endorsement between these assessment formats when relying on caregiver report, with agreement and concordance estimates ranging greatly from low to high (e.g., Allen et al., 2012; McGuire et al., 2024a). However, the factors explaining these differences are not well understood, as minimal research has evaluated why discrepant reporting might occur. Understanding why such discrepancies occur can help provide guidance to the field on improving ALE measurement and potentially address weaknesses inherent within each measurement format.

In one of a very limited number of studies available with young children, Glackin and colleagues (2019) surveyed low-income caregivers of preschool age children to examine the role of caregiver characteristics in explaining these discrepancies. The authors found that caregiver psychopathology (e.g., distress) and low education were associated with lower concordance between assessment methods when asking about children’s ALE exposure (e.g., sudden loss of a loved one). Relatedly, Wagner and colleagues (2006) found that caregivers’ anxiety symptoms, but not depressive symptoms or cognitive vulnerability, were associated with overreporting of ALE severity scores on a questionnaire relative to an interview, but none of these factors were associated with differences in endorsement of ALE types between these measures. Aside from the studies by Glackin et al. and Wagner et al., most research on ALE assessment formats discrepancies has come from adults reporting about their own ALEs, often looking at the individual factors of the reporter in relation to agreement. For example, studies on adults have revealed an association between adult mental health challenges (e.g., distress, posttraumatic stress symptoms) and format discrepancies when reporting on ALEs (e.g., Van Geizan et al., 2005).

However, there are several factors in the adult self-report literature of ALEs that have yet to be examined when relying on adult (i.e., caregiver) report of a child’s ALE exposure. One domain is caregiver beliefs of assessment formats. For example, evidence from the adult literature suggests that privacy or confidentiality beliefs may relate to ALE reporting differences between interview and questionnaire formats. Specifically, questionnaires are sometimes thought to provide more anonymity and privacy compared to interviews (e.g., Decker et al., 2011; DiLillo et al., 2006). For example, Kubiak and colleagues (2012) compared response rates for ALEs between an interview and anonymous survey in a sample of predominately Black female adults, finding that physical and sexual assault rates were approximately twice as high in the anonymous questionnaire compared to the face-to-face interview. However, contrasting evidence has also been published suggesting interview and questionnaire formats produce similar levels of perceived confidentiality. For example, Reddy and colleagues (2006) examined adult participants’ willingness to report on sensitive topics (e.g., substance use, abuse) using a questionnaire, phone interview, face-to-face interview, and automated phone approach, finding similar perceived confidentiality between the in-person questionnaire and interview. Beyond confidentiality, research has also demonstrated that perceived positive experiences and costs/benefits may also relate to differences in reporting in the context of a research study (DePrince & Chu, 2008; Jaffe et al., 2015; Legerski & Bunnell, 2010).

## Present Study

Collectively, there has been minimal research examining potential factors underlying agreement between assessment approaches for caregiver reporters of children’s ALEs. However, research on this issue is critical to improving evidence-based assessment practices of ALEs for children, as well as to better serve children and families exposed to ALEs. Further, while the adult literature can provide auxiliary evidence for how adults might respond when reporting about their child’s ALEs, evidence suggests that the application of this literature is limited. For example, Lewinsohn et al. (2003) compared reports from adults on interview and questionnaire approaches for ALEs occurring to the self and close others (e.g., spouse/partner, close relative, or close friend), finding that approximately 20% of reported events that occurred to other close individuals were reported on both the questionnaire and interview formats. The level of agreement between formats for events occurring to the close individuals was much less compared to the amount of agreement when reporting about themselves, suggesting that reporting behaviors between formats may vary depending on who is being reported on, even for other close family members.

To address these knowledge gaps, the current study examined several caregiver specific factors identified in the adult and child ALE assessment literature that may contribute to lack of agreement between face-to-face interviews and paper-pencil questionnaires. This included examining caregiver assessment belief differences between ALE formats, as well as examining psychological concerns and individual level demographics. This was achieved by administering caregivers a similar and comprehensive ALE assessment that inquired about 50 different types of ALEs in both interview and questionnaire formats. Based on the adult self-report literature, it was hypothesized that confidentiality/privacy beliefs and mental health symptoms would demonstrate a relation with agreement, such that caregivers who had greater differences in their confidentiality/privacy belief scores and higher reported mental health symptoms would demonstrate lower agreement in the ALE reporting between measures. Given the limited and mixed literature in this domain, there were no specific hypotheses regarding the direction of this relation as it relates to over or under reporting on a certain ALE assessment format.

## Method

### Participants

Participants were 57 caregivers of preschool and school-age children, recruited from a large midwestern county in the United States. Participants were connected to the research project through local agencies and organizations serving families from predominately low-income backgrounds (e.g., local food support services and Head Start). The following eligibility criteria were utilized to ensure caregivers could reliability and ethically participate in the study: (a) have a child 3–9 years of age, (b) be the legal guardian of the child, (c) caregiver’s and child’s primary language is English, and (d) have no self-reported developmental or autism spectrum disorder diagnosis for the child or caregiver. Sixty caregivers who enrolled in the study. Three caregivers were excluded from data analysis for not completing the second time point. Caregivers’ mean age was 33.72 years (*SD* = 6.97) and most identified as biological mothers (96.5%). Children were on average 6.11 years old (*SD* = 1.60, range: 3–9), and there was a slight majority of male children (57.9%). Most caregivers (61.4%) and their child (59.6%) identified as Black or African American, followed by white (caregiver = 29.8%, child = 26.3%) and then multiracial (caregiver = 3.5%, child = 10.5%) or Asian for caregivers (3.5%). According to the U.S. Department of Health and Human Services (2021) federal poverty guidelines using yearly family income and individuals in the household, over half (54.3%) of families who participated were below the federal poverty level.

### Measures

#### Demographics

Caregivers completed a demographics form that included information on their and their child’s age, sex, family income, living situation, race, and ethnicity.

#### ALE Assessment Beliefs

Caregivers completed a modified version of the Reactions to Research Participation Questionnaire for Parents (RRPQ-P; Kassam-Adams & Newman, 2002, 2005) to assess for beliefs about completing the ALE assessments. The RRPQ-P measures views about participating in research with four subscales (12 items total; 3 items each subscale): Positive Appraisals, Negative Appraisals, Consent/Privacy Issues, and Understanding of Consent Form. These subscales form two composite scales for Total Positive Appraisal (Positive Appraisals, Negative Appraisals) and Trust/Information (Consent/Privacy Issues, Understanding of Consent Form). Caregivers responded to each item using a five-point Likert scale from 1 (“*No: I strongly disagree*”) to 5 (“*Yes: I strongly agree*”). The present study used two similar but modified versions (interview and questionnaire versions) of the measure. Each measure used the same questions and same question structure. However, at each assessment session, the words were modified to ask specifically about the administered ALE assessment at each assessment session (e.g., for a ALE interview Consent/Privacy question - “*The things I said today in the interview will stay private*;” for a ALE questionnaire Consent/Privacy question - “*The things I said today in the questionnaire will stay private*”). The RRPQ-P has demonstrated satisfactory reliability and validity in studies involving caregivers with children exposed to ALEs (Kassam-Adams & Newman, 2002). Internal consistency measurements using Cronbach’s alpha (α) and McDonald’s Omega (ω) for the current study were below satisfactory for some but not all subscales: Negative Appraisals- interview (α = 0.46, ω = 0.62), questionnaire (α = 0.56, ω = 0.57); Positive Appraisals- interview (α = 0.59, ω = 0.70), questionnaire (α = 0.64, ω = 0.71); Consent/Privacy Issues- interview (α = 0.65, ω = 0.72), questionnaire (α = 0.70, ω = 0.73); Understanding of Consent Form- interview (α = 0.75, ω = 0.80), questionnaire (α = 0.83, ω = 0.86). Because of the low internal reliability measurements for the Negative Appraisals subscale across both format types, this individual subscale was not evaluated independently in the analyses. Internal consistency measurements were satisfactory for the composite scales: Total Positive Appraisals- Interview (α = 0.73, ω = 0.73), questionnaire (α = 0.73, ω = 0.74); Trust/Information- Interview (α = 0.81, ω = 0.83), questionnaire (α = 0.85, ω = 0.86).

#### Caregiver Mental Health

To examine caregivers’ mental health symptoms, the Brief Symptom Inventory (BSI; Derogatis, 1993) was administered at the first time point of the study. The BSI is a 53 item self-report measure of symptomology across several different domains of mental health concerns. The current study used the Global Severity Index (GSI) composite score. Participants responded using a five-point Likert scale from 0 (“*Not at all*”) to 5 (“*Extremely*”) based on their symptoms over the last seven days. The BSI has demonstrated satisfactory psychometric properties in adults (e.g., Boulet & Boss, 1991; Hoe & Brekke, 2009). The composite GSI scale demonstrated satisfactory internal consistency in this study (α = 0.96, ω = 0.96).

#### Child’s ALE Exposure

Child’s lifetime ALE exposure history was obtained using modified versions of the PAIR Intergenerational Trauma Measure (PAIRIT), which was an assessment instrument created for the PAIR project through combining multiple ALE assessments to ensure a comprehensive analysis of all types of ALEs (for more information, please see Huffhines et al. [2021] or McGuire et al. [2024a]). The PAIRIT assesses for 50 different types of ALEs, such as exposure to direct and indirect violence, illness, natural disasters, and household dysfunction. Caregiver were asked to indicate yes (1) or no (0) whether their child experienced each event. The PAIRIT also assessed for ALE characteristics (e.g., age of onset, frequency). However, due to sample size and endorsement among the 50 different types of events, only type and polyvictimization of exposure was used in the current study. There were two forms of the PAIRIT- an interview and questionnaire format. Each format included the same 50 ALE types using a similar question structure. For the questionnaire, all questions were administered in the same set order using a paper-pencil packet. Participants were monitored by research staff during completion of the questionnaire and permitted to ask the research staff questions if needed as they completed the packet. The interview format used a semi-structured design at the beginning of the interview, which is commonly used in ALE exposure interviews (Harkness & Monroe, 2016). That is, caregivers were first asked to provide a list of events the child had experienced. The interviewer then started with these events by asking for follow up information using an open-ended prompt (e.g., *“Please tell me as much information as you can about this event”*). Following completion of the caregiver’s open-ended list of events, the interviewer then proceeded through the remaining types of events not initially discussed until all 50 events were assessed. Of note, all interviews were conducted with the same interviewer, who identified as a white, non-Hispanic, cisgender male and was a mental health professional with experience serving ALE exposed populations.

### Procedures

All procedures were approved by the institutional review board of the first author’s institution. There were two time points in the current study, which used a repeated-measures within-subjects design. Following participant recruitment and screening, participants were scheduled for the first data collection session at an accessible community location (e.g., local community event space). At the first session, participants completed consent procedures and then were administered a battery of study questionnaires via an online survey with a laptop computer, which for the current study consisted of the demographics and BSI measures. Next, participants were administered the first ALE assessment type. To ensure counterbalancing, participants were randomly assigned at sign up to begin with either the interview or questionnaire (*n* = 29 began with the questionnaire, *n* = 28 began with the interview). Immediately after completing the ALE assessment, participants completed the RRPQ-P that corresponded with that version of the ALE assessment. Last, the participants completed a debriefing process with a member of the research team and were then scheduled for their second data collection session. Because of concerns about influence on reporting behaviors and the current study being part of a larger research, participants were not told that part of the study was examining their responses to the ALE assessment.

The second data collection occurred 7 to 21 days after the first data collection session, which was an appropriate timeframe to reduce the possibility of reporting fatigue or memory recall bias (e.g., Thabrew et al., 2012). The second data collection followed a similar procedure to the first data collection. Participants again engaged in a consent form review process with a member of the research team, followed by completion of the second form of the ALE assessment. Again, immediately following the ALE assessment, participants completed the corresponding version of the RRPQ-P. At the end of the second session, participants completed a second debriefing session with a member of the research team and were provided financial compensation for their participation. Of note, all data collection occurred during the COVID-19 pandemic. Thus, all research study personnel and participants wore facemasks during all data collection activities.

### Data Analysis

All analyses were conducted in R Software- Version 4.3.0 (R Core Team, 2023). Descriptive information for participants and the main variables of interest were generated. Difference scores between each assessment format were calculated for the assessment format belief variables by subtracting the questionnaire values from the interview values (e.g., subtracting the mean positive appraisal score of the questionnaire RRPQ-P from the mean positive appraisal score for the interview RRPQ-P). This allowed for the examination of overall differences in beliefs between each assessment format. Additionally, a total agreement score for the 50 different types of ALEs was calculated by summing together the number of similar responses for all 50 ALE types between the questionnaire and interview (i.e., if the item related to exposure to community violence was endorsed on both the interview and questionnaire format, or not endorsed on both the interview and questionnaire, the participant would receive a 1 for that ALE). Additionally, for each participant, an unweighted Cohen’s kappa coefficient was generated based on the 50 ALE types between each assessment format to examine reporting concordance (Cohen, 1968; Tang et al., 2015). The correlations between the primary variables in the current study were also generated.

Next, a series of paired-samples *t*-tests were calculated to examine differences in reporting between the interview and questionnaire regarding ALE sum polyvictimization scores. Moreover, paired-samples *t*-tests were used to compare the mean values for the composite and subscales of the RRPQ-P for each assessment approach to evaluate differences in beliefs between each assessment format. The *t*-statistics were examined for significance at *p* <.05. Lastly, a series of multiple linear regression models were generated using ordinary least squares to examine factors that may contribute to agreement between the questionnaire and interview ALE assessments. The sum agreement score was used as an outcome variable in the two examined models. For each model, caregiver’s race congruency with the interviewer (0 = interview congruent [i.e., white)] and 1 = non-congruent), federal poverty level, and BSI-GSI raw score were included in each model as predictors. In addition to these three variables, one of the two RRPQ-P composite scales (i.e., Total Positive Appraisal and Trust/Information) was also included in the model. An a-priori power analysis indicated that a sample size of 57 for a multiple linear regression model with four predictors would be able to determine a medium size effect with a power level of 0.60 or a large size effect with a power level of 0.95. The regression models were evaluated for regression coefficient significance using *p* <.05 and both the *R*^2^ and Adjusted *R*^2^.

## Results

### ALE Exposure

The descriptive information for the primary variables of interest and participants is in Table 1. On average, caregivers reported exposure to multiple types of ALEs for their child, as indicated by a mean polyvictimization score for the interview of *M* = *9.42* (*SD* = 4.49) and for the questionnaire of *M* = *8.61 (SD* = 5.62). Greater than 94% of children were reported to have been exposed to two or more types of ALEs based on either the interview or questionnaire format. There was a non-significant difference in polyvictimization scores between assessment formats, *t*(56) = 1.35, *p* =.18. The percentages of endorsement to all 50 types of events for both the interview and questionnaire measures are presented in Supplementary Table S1. To check for possible order effects between the questionnaire and interview, scores were compared for the polyvictimization total score according to the starting format. All paired sample *t*-tests were non-significant (i.e., all *t*s < 0.79, all *p*s > 0.43). Concordance estimates using Cohen’s kappa ranged from − 0.03 to 1.00 per participant for the 50 types of events. There was 1 participant in the poor range (i.e., κ < 0.00), 11 participants in the slight to fair range (κ = 0.00–0.40.00.40), 37 participants in the moderate to substantial range (κ = 0.40–0.80.40.80), and 8 participants in the almost perfect to perfect range (κ = 0.81 − 1.00).

**Table 1:** Participant demographics

**Child Demographics**	**Mean** **(*SD*)** **or %**	**Median**	**Range**	**Possible Range**
Child Age (years)	6.11 (1.60)	6.35	3–9	3–9
Child Sex (% female)	42.1%			
Child Race				
NA/AN	0.0%			
Asian	3.5%			
Black/African American	59.6%			
NH/OPI	0.0%			
White	26.3%			
Mixed Race/Multiracial	10.5%			
Child % Hispanic/Latino	7.0%			
**Caregiver Demographics**	**Mean (** ***SD*** **) or %**	**Median**	**Range**	**Possible Range**
Caregiver Age	33.72 (6.97)	32.00	22–61	18.00+
Caregiver Relationship				
Biological Mother	96.5%			
Biological Father	1.8%			
Grandmother	1.8%			
Caregiver Race				
NA/AN	0.0%			
Asian	3.5%			
Black/African American	61.4%			
NH/OPI	1.8%			
White	29.8%			
Mixed Race/Multiracial	3.5%			
Caregiver % Hispanic/Latino	3.5%			
Family FPL				
50% below FPL	36.8%			
50% to 100% of FPL	17.5%			
100% to 150% of FPL	12.3%			
150% to 200% of FPL	8.8%			
Above 200% of FPL	24.6%			
**Caregiver Mental Health**	**Mean (** ***SD*** **) or %**	**Median**	**Range**	**Possible Range**
BSI- Global Symptom Index (GSI*)*	1.52 (0.51)	1.36	1.00–3.11.00.11	1.00–5.00

FPL = Federal Poverty Level. NA/AN = Native American/Alaska Native. NH/OPI = Native Hawaiian or Other Pacific Islander. SD = Standard deviation

### Research Participation Beliefs

The descriptive information for ALE format belief measures for both the interview and questionnaire assessment formats is presented in Table 2. Overall, composite and subscale scores on the RRPQ-P were similar between the interview and questionnaire format. This included similar reports (i.e., non-significant difference scores) for the Total Positive Appraisal and Trust/Information composite scales, as well as on the Positive Appraisals and Consent/Privacy Issues subscales. The only exception was for the Understanding the Consent Form subscale, which demonstrated a significant mean difference between the formats, *t*(56) = 1.97, *p* =.05. This suggested that participants tended to have stronger beliefs in their ability to exercise their research rights when completing the interview compared to the questionnaire. A post-hoc analysis used paired sample *t*-tests with each individual item of this subscale suggested that the difference was largely driven by RRPQ-P item 11 (“*I knew I could stop the [interview/questionnaire] at any time today*”), *t*(56) = 2.25, *p* =.03. This indicated that participants tended to have a better understanding of their ability to stop the interview, compared to stopping the questionnaire. All the other items on this scale demonstrated either marginally or non-significant differences between formats. 

**Table 2:** Adverse life event (ALE) assessment beliefs

**ALE Assessment Belief Variables**	Interview Format			Questionnaire Format			Format Comparisons		
	**Mean (** ***SD*** **)**	**Median**	**Range**	**Mean (** ***SD*** **)**	**Median**	**Range**	**Mean Difference** ^**¢**^	***t*** **-test** ^§^	**Cohen’s** ***d***
*RRPQ-P Composite Scales*									
Total Positive Appraisal	4.47 (0.61)	4.67	2.33–5.00	4.34 (0.47)	4.33	3.33–5.00	0.11	1.73	− 0.23
Trust/Information	4.56 (0.50)	4.67	3.50–5.00	4.47 (0.61)	4.67	2.33–5.00	0.08	1.09	− 0.14
*RRPQ-P Subscales*									
Positive Appraisals	4.38 (0.55)	4.33	2.67–5.00	4.28 (0.59)	4.33	2.67–5.00	0.11	1.30	− 0.17
Consent/Privacy Issues	4.60 (0.55)	5.00	2.33–5.00	4.61 (0.57)	5.00	2.33–5.00	− 0.02	− 0.21	0.03
Understanding of Consent Form	4.52 (0.60)	5.00	3.00–5.00	4.33 (0.78)	4.33	1.00–5.00	0.19	1.97*	− 0.26

Total *N* = 57. * = *p* <.05. § = paired samples *t*-test analyses. ^**¢**^ **=** Mean differences were calculated by subtracting the questionnaire values from the interview values. ALE = Adverse life event. *M* = mean, *SD* = standard deviation

## Correlations among Study Variables

The correlations among the primary variables are presented in Table 3. All the composite and subscales of the RRPQ-P across formats were significantly correlated, except for the correlation between the Interview RRPQ-P- Consent and Questionnaire RRPQ-P- Total Positive Appraisal variables. FPL Status was also significantly, negatively correlated with caregiver mental health symptoms (i.e., BSI-GSI) and exposure to ALEs on the interview and questionnaire assessments, and it was significantly, positively correlated with concordance between the assessment formats. Caregiver mental health was significantly, positively correlated with exposure to ALEs (for both interview and questionnaire scores), and it was also significantly, negatively correlated with the Positive Appraisal composite scale for both the interview and questionnaire format beliefs.

**Table 3:** Correlations between primary study variables

Study Variables	1	2	3	4	5	6	7	8	9	10	11	12	13	14	15	16	19
1. Child’s Age																	
2. Child’s Sex	0.14																
3. Federal Poverty Level Status	− 0.02	− 0.19															
4. Brief Symptoms Inventory-GSI	0.06	− 0.11	− 0.15*														
5. Questionnaire ALE Polyvictimization	− 0.04	0.06	− 0.13*	0.38*													
6. Interview ALE Polyvictimization	0.15	0.29	− 0.26*	0.39*	0.62*												
7. Type of ALE Cohen’s Kappa	0.04	− 0.08	0.29*	0.07	0.23	0.06											
8. Type of ALE Total Agreement Score	− 0.06	− 0.25*	0.39*	− 0.12	− 0.35*	− 0.47*	0.66*										
9. Interview RRPQ-P- Negative	0.08	0.07	0.10	0.24*	0.12	0.12	0.09	0.06									
10. Interview RRPQ-P-Positive	− 0.06	0.01	− 0.30	− 0.07	0.05	0.02	− 0.19*	− 0.18	− 0.55*								
11. Interview RRPQ-P Privacy	− 0.01	0.13	− 0.17	− 0.06	0.05	0.04	0.04	0.01	− 0.38*	0.53*							
12. Questionnaire RRPQ-P- Privacy	− 0.11	0.10	0.14	− 0.01	0.03	0.09	0.13	0.11	− 0.39*	0.12*	0.39*						
13. Interview RRPQ-P- Consent	0.07	0.08	0.20	0.07	0.19	0.13	− 0.03	− 0.11	− 0.27*	0.17*	0.48*	0.34*					
14. Questionnaire RRPQ-P- Consent	− 0.07*	0.19	0.15	0.04	0.04	0.05	0.06	0.04	− 0.23*	0.02*	0.19*	0.63*	0.48*				
15. Interview RRPQ-P- Total Positive App.	− 0.08	− 0.03	− 0.24	− 0.17*	− 0.04	− 0.05	− 0.17	− 0.01	− 0.86*	0.90*	0.52*	0.28*	0.25*	0.14*			
16. Questionnaire RRPQ-P- Total Positive App.	− 0.07	0.10	0.01	− 0.16*	− 0.20*	− 0.03	− 0.03	0.06	− 0.52*	0.39*	0.17*	0.55*	0.08	0.36*	0.51*		
17. Interview RRPQ-P- Trust/Information	0.04	0.12	0.03	0.01	0.14	0.10	0.00	0.08	− 0.38*	0.40*	0.85*	0.42*	0.87*	0.40*	0.44*	0.14*	
18. Questionnaire RRPQ-P- Trust/Information	− 0.10	0.17	0.16	0.02	0.04	0.07	0.10	0.16	− 0.32*	0.07*	0.31*	0.87*	0.47*	0.93*	0.22*	0.49*	0.45*

Total *N* = 57. * = *p* ≤.05. Bold numbers also indicate *p* ≤.05. ALE = Adverse life event. GSI = General Symptom Index. RRPQ-P = Reactions to Research Participation Questionnaire- Parent

### Multiple Linear Regression Model Analyses

The multiple linear regression model outcomes are presented in Table 4. For both models, caregiver race congruence and BSI-GSI scores did not significantly predict agreement between interview and questionnaire formats, nor did the RRPQ-P Total Positive Appraisal difference score in Model 1 or the RRPQ-P Trust/Information differences score in Model 2. However, federal poverty level status was significantly positively associated with total agreement in both models (Model 1: *B*[*SE*] = 1.01 [0.33], *p* <.01); Model 2: *B*[*SE*] = 0.98 [0.33], *p* <.01), suggesting that caregivers from higher poverty levels, relative to lower poverty levels, tend to have greater agreement between formats regarding type of ALE exposure.

**Table 4:** Models examining differences between the interview and questionnaire format

Models Tested	Total ALE Type Agreement
**Model 1**	**Beta Coefficients (** ***SE*** **)**
Poverty Level	1.01 (0.33)**
Caregiver Race Congruence	1.02 (0.98)
BSI-GSI	− 0.52 (0.89)
RRPQ Total Positive Appraisal Diff	0.24 (1.01)
*R* ^2^	0.17
Adjusted *R*^2^	0.11
**Model 2**	**Beta Coefficients (** ***SE*** **)**
Poverty Level	0.98 (0.33)**
Caregiver Race Congruence	0.98 (0.99)
BSI-GSI	− 0.53 (0.89)
RRPQ Trust/Information Diff	− 0.10 (0.78)
*R* ^2^	0.17
Adjusted *R*^2^	0.11

Total *N* = 57. **= *p* ≤.01. Outcome variable for both models was Total ALE Type Agreement. SE = Standard error. ALE = Adverse life event. Diff. = Difference score. BSI-GSI = Brief Symptom Inventory- Global Severity Index. For Caregiver Race Congruence- 0 = Congruent with interviewer (i.e., white), 1 = Not congruent with interviewer

## Discussion

Literature on caregiver report of young children’s ALE exposure has revealed discrepancies between interviews and questionnaire assessment formats (e.g., Allen et al., 2012; McGuire et al., 2024). However, there has been little research focused on identifying factors that might explain why these discrepancies occur. The present study addressed this research gap by examining multiple caregiver factor domains (e.g., caregivers’ assessment beliefs, mental health, demographics) in relation to agreement in reports of type of ALE exposure between face-to-face interviews and paper-and-pencil questionnaires for caregivers of young children. The primary findings were that participants were more likely to believe they could exercise their research rights during the interview format relative to the questionnaire format. Caregiver mental health, race congruence with the interviewer, and participation beliefs were not significantly associated with total agreement, but participants from lower federal poverty levels tended to demonstrate lower agreement between formats, compared to participants from higher income levels.

### General Agreement between ALE Assessment Formats

Overall, the majority of participants demonstrated similar reports between the interview and questionnaire assessment ALE methods (i.e., Cohen’s kappa coefficients > 0.40 and no significant difference in ALE polyvictimization total score between interview and questionnaire). However, similar to prior studies (e.g., Glackin et al., 2019; Wagner et al., 2006), concordance varied greatly from very poor to perfect among participants in the present study. The present data expanded on this prior research by demonstrating that this is the case across a more comprehensive inventory of ALEs than has been included in other similar studies (e.g., Wagner et al., 2006). For example, this included differences in reporting among events that are likely to be experienced by families from low-income levels and be more chronic rather than single instance events (e.g., significant reduction in living standards, forced to leave home due to financial concerns).

### Beliefs about ALE Assessment Formats Relating to Agreement

The primary purpose of the present study was to determine what factors might help explain agreement between interview and questionnaire formats. One domain that had yet to be examined in relation to caregiver report of young children’s ALEs was caregivers’ beliefs about the assessment formats. As it relates to the current study’s hypothesis regarding confidentiality and privacy being associated with agreement between formats, this hypothesis was not supported. This was observed through non-significant relations with ALE agreement in the regression models, as well as no observed relations in the correlations and *t*-test analyses. This was also the case for the belief variables related to positive experiences in the regression model.

While participation beliefs did not appear to be strongly associated with level of agreement between the ALE assessment methods, some differences did emerge regarding general beliefs about completing each format type. This was observed with the overall format mean score comparisons related to caregiver beliefs for the Understanding the Consent Form subscale of the RRPQ-P. This finding suggested that there was on average more understanding of participants’ ability to exercise their rights during completion of the interview compared to the questionnaire. This difference in consent form and participant rights beliefs was reported in the current study, despite participants receiving the same consent procedures prior to each ALE assessment at each data collection session. One reason for this finding may be that participants felt more comfortable asking clarification questions or being willing to stop the assessment if they wanted during the interview since they were already engaged in conversation with the interviewer during this assessment format. This contrasts with completing the questionnaire, which was largely independent (although participants could ask questions and stop if needed). As compared to questionnaires, interviews are thought to allow interviewees and the interviewers greater opportunity to clarify concerns and ask questions, as well as build rapport, which in turn may relate to willingness to express interest in stopping or not answering a question (e.g., Harkness & Monroe, 2016).

### Caregiver Characteristics Relating to ALE Assessment Method Agreement

In addition to caregiver beliefs, several other individual level caregiver factors were examined in relation to reporting differences. Overall, emotional and behavioral concerns did not appear to be associated with differences in reporting behaviors between the questionnaire and interview formats, which was in contrast with the study’s hypothesis. These findings contrast with some previous literature suggesting that general and anxiety specific mental health challenges may contribute to caregiver reporting differences between an interview and questionnaire ALE assessment (e.g., Wagner et al., 2006; Glackin et al., 2019). Post-hoc analyses with the depression and anxiety subscales of the BSI in the regression models (as opposed to the BSI-GSI composite scale) demonstrated non-significant associations as well. One explanation that may explain some of the differences between Glackin and colleagues’ findings and the current study for overall mental health could be related to the types of ALEs examined. Glackin and colleagues found reporting differences related to caregiver mental health in relation to specific forms of ALE, including sudden loss of a loved one. The present study examined a broader list of events and agreement between all forms of ALEs. Thus, it may be the case that caregiver mental health challenges are uniquely related to reporting differences among certain types of ALEs.

In contrast to some prior research with adults reporting about their own ALE exposure (e.g., Glackin et al., 2019), caregiver and interviewer race congruence did not appear to be related to reporting differences between the ALE assessment formats. However, another important pattern observed in the current study was the relation between federal poverty level status and ALE reporting agreement, which suggested that those individuals from lower poverty levels tended to demonstrate less concordance for reporting on ALEs compared to higher levels. One explanation for this finding may be related to overall rates of ALE exposure. As a result of systematic injustices and persistent barriers to services, families with financial difficulties are often at-risk for exposure to more ALEs, compared to middle- and upper-class families (Slopen et al., 2016; Walsh et al., 2019). This also appeared to be the case in the current study, as being higher or above the federal poverty level was negatively correlated with exposure to more types of ALEs. Thus, it may be that caregivers from low-income households had difficulty remembering a larger and more diverse set of ALEs their children experienced between formats. It is also worth noting that many items on the current study’s ALE assessment were related to financial difficulties (e.g., forced to leave home, reduction in standard of living). However, this is only one possible explanation for this finding. Follow up research is needed in this area that further evaluates which types of ALEs were more likely to be reported differently and why. For example, this might include research to explore whether there is purposeful omission of certain items as a result of factors such as social disability or less investment in contributing to research, which have been documented in low-income populations (e.g., Tourangeau & Yan, 2007). It is also important to note that federal poverty level also demonstrated a significant correlation with concordance in the correlation analyses. This may also suggest that while overall “scores” or ALE variables are similar between assessments for families from low-income backgrounds, the types of events that go into generating these scores may be different based on the assessment format used. This speaks to the importance of properly assessing families from low-income backgrounds to ensure an accurate picture of ALE exposure is captured, as such assessments may be especially critical to ensuring appropriate service delivery.

### Limitations

The present study has several limitations. First, the study’s small sample size limited the types of analyses that could be conducted and was associated with low power to detect medium-to-small effects in the regression models and group comparisons. This may have also been associated with low internal consistency and thus low measure reliability, as internal reliability estimates for some of the RRPQ-P subscales were below an acceptable range. However, it is important to note that the composite scales were in the acceptable range. Second, the study did not provide an assessment of posttraumatic stress symptoms, which have been found in prior research on caregiver report and the general adult assessment literature to influence ALE reporting behaviors (e.g., Glackin et al., 2019). Third, the use of facemasks due to the COVID-19 pandemic may have influenced reporting behaviors, as previous research in other related assessment fields has found initial evidence that facemasks can influence reporting of sensitive or distressing topics (e.g., Biermann et al., 2021). Additionally, a limitation is that the study did not include questions directly assessing maltreatment exposure. Questions were included on removal from the home for these types of experiences, but direct experiences to these ALEs was not. Given previously documented methodological, cultural, and other systemic factors associated with disclosure of maltreatment specific experiences (e.g., Alaggia et al., 2019), this may have influenced concordance and differences in reporting between the formats. Relatedly, while the ALE assessments were comprehensive, they did assess for 50 different types of ALEs. The length of these assessments may have negatively impacted reporting, as longer assessments/questionnaires may be related to lower response rates (e.g., Rolstad et al., 2011). Another limitation is that all interviews were conducted by the same interviewer. Although this approach may have reduced common-method variance, this did not allow for the current study to explore possible interviewer influences on caregiver reporting beyond broad interview-interviewee race congruence (e.g., inability to evaluate possible gender differences between interview-interviewee may related to agreement between methods, which has been shown to influence adult reporting behaviors on sensitive topics, such as one’s own exposure to ALEs; Wilson et al., 2002).

### Future Research and Clinical Recommendations

The present data provide evidence in support of several research and clinical recommendations when working with children and families exposed to ALEs. First, the findings regarding differences in reporting between formats echoes calls in the literature to use dual assessment approaches to ALE exposure in children to address format limitations and thus potentially increase assessment accuracy (e.g., Glackin et al., 2019; McGuire et al., 2024b). The present data and literature at large appear to suggest that one assessment method or source may provide an incomplete picture of children’s ALE history. This may be especially true for children and families from low-income and racial/ethnic minority populations, who may be exposed to more types of ALEs and experience significant barriers to access services that address the negative consequences of ALE exposure. Thus, using complementary assessment formats may help ensure all types of events are captured.

Further, using two formats would also allow for additional testing of differences between formats and why such differences may emerge, which is an understudied area in ALE assessment as it relates to caregivers serving as reporters. One unique approach to the present study was associated with the design of the interview and questionnaire format, such that each version of the ALE assessment followed a similar question and answer structure. This contrasts with prior studies on caregivers’ report of their child’s ALE exposure, which have tended to rely on different measures for the interview and questionnaire format (e.g., Allen et al., 2012; Glackin et al., 2019; Wagner et al., 2006). Although an intuitive recommendation, it is recommended that future development of ALE assessments for use in research and clinical work use interview and questionnaire formats that mirror each other. This study’s findings related to general lack of differences in caregiver beliefs about each assessment and the general lack of association between assessment beliefs on reporting differences further emphasize this recommendation. Designing ALE assessments in this way may help reduce discrepancy in reporting between formats by reducing the chance of caregivers’ experiences/beliefs (e.g., privacy concerns) negatively influencing concordance.

The only notable difference observed in the present study was in participants’ views of their research rights, which tended to show that participants more strongly agreed with their rights as research participants while completing the interview compared to the questionnaire (e.g., ability to take breaks, refuse to answer questions). Based on this finding, it is recommended that the mirroring in questionnaire and interview format administration extend beyond the measure itself to include similar consent form and administration methods for both formats. That is, researchers and clinicians follow the same procedures when administering both an interview and questionnaire. As it relates to questionnaire formats, researchers and clinicians may need to build in verbal check-ins with individuals or scheduled breaks, similar to what might naturally occur when completing an interview. Doing so may reduce levels of distress a participant experiences, as well as reduce overall anxiety related concerns. In turn, this may positively influence reporting behaviors and increase concordance between assessment formats (Glackin et al., 2019). These approaches may be especially important when working with patients or participants from racial/ethnic minorities and financially challenged populations, given documented concerns of misinformation related to research participation among these populations (e.g., Quinn et al., 2012).

It is important to recognize that such recommendations related to using two assessment formats in a single study often require more resources for researchers and more effort from participants. However, accurate ALE assessment is a necessary consideration, especially when researchers are examining ALE exposure in relation to children’s functioning or another variable. It is recommended that researchers plan for such approaches prior to study development. For example, these types of approaches can be written into grant proposals, helping to ensure that researchers will have the necessary financial and personnel resources to administer two types of ALE assessments. There also exist a number of well-conducted review articles in the literature that examine and compare the logistical considerations of various ALE assessment tools, so that researchers can select a dual assessment method that may fit best the needs of their study (e.g., Eklund et al., 2018; Oh et al., 2018). Based on the findings from the current study, it does not appear that caregivers experience much burden from needing to complete two similar but different ALE assessments, as the majority of caregivers had positive appraisals of both formats, which did not change based on whether the assessment was administered first or second. This partially aligns with findings from the child report literature, such that youth report minimal negative reactions to completing detailed assessments of their ALE exposure, despite hypothesized concerns about overburdening or “retraumatizing” them (e.g., Knight et al., 2000: Zajac et al., 2011).

In addition to these methodological recommendations, there are also some important recommendations for future research to further understand differences in caregiver reports of their child’s ALEs according to assessment format. One primary recommendation is to explore possible interactions between caregiver factors that may relate to agreement. For example, it may have been the case that there were important moderating influences between mental health factors and caregiver beliefs, such as how certain mental health challenges may be exacerbated by whether the caregiver feels comfort or privacy while completing an ALE assessment. Another area of research in caregiver reporting differences might be to explore in greater depth how type or class of ALE relates to the observed relations in the current study. For example, Glackin and colleagues (2019) and McGuire and colleagues (2024a) found differences in amount of concordance among caregivers’ reporting between interview and questionnaire ALE forms depending on the type or class of ALE. It may be the case that caregiver factors, such as mental health and privacy beliefs, become more pronounced depending on the type of ALE examined (e.g., relational vs. non-relational ALEs between the child and caregiver). Moreover, given the findings on beliefs of the consent form and research rights, future research in this field should also seek to examine the influence of context or settings on reporting discrepancies. For example, this might include examining how best to review consent forms with participants before conducting a study on ALE assessment. Overall, future research should seek to not just examine caregiver factors in isolation, but also consider how these variables might interact with other one another, as well as child and broader assessment characteristics of ALEs. Future research with larger heterogeneous samples of caregivers could build on the current study’s research by evaluating such combinations of factors.

## Conclusions

Taken together, the present study provides an important step forward in understanding why discrepancies may emerge between assessment formats when relying on caregivers to provide information on their child’s ALEs. This was the first study to explore caregivers’ beliefs related to completing an interview and questionnaire version of an ALE assessment. Future research is needed to further explore these variables, as well as their interaction with other known contributors to lack of concordance (e.g., caregiver mental health), to understand under what circumstances differences in reporting may emerge. The findings further emphasize the importance of using multiple methods or sources of information to ensure a comprehensive and accurate account of children’s ALE exposure history is obtained in both research and clinical practice.

## Supplementary Information

Below is the link to the electronic supplementary material.

ESM 1 (DOCX 45.6 KB)
