# Supplementary material for: Improving the Assessment of Childhood Adversity: Factors Influencing Questionnaire and Interview Method Concordance
Source: J Child Adolesc Trauma. Author manuscript; Available in PMC 2026 Mar 24. (PMC13004759; doi:10.1007/s40653-026-00817-2)
Supplement: Supplement [file NIHMS2155077-supplement-Supplement.docx]

| **Supplementary Table S1.**  *Percent Endorsement of each Adverse Life Event Type* | | |
| --- | --- | --- |
| **Adverse Life Event Type** | **% Endorsed on Questionnaire** | **% Endorsed on  Interview** |
| Moved to a new home | 71.9% | 77.2% |
| Changed childcare provider | 52.6% | 86.0% |
| Caregivers separated | 49.1% | 47.4% |
| Caregiver admitted to a hospital | 49.1% | 57.9% |
| New child moved into the home | 42.1% | 49.1% |
| Close adult died | 40.4% | 43.9% |
| Heard about something terrible | 36.8% | 31.6% |
| Caregiver experiences mental illness | 36.8% | 49.1% |
| Reduced standard of living | 31.6% | 15.8% |
| Caregiver or close individual arrested | 31.6% | 28.1% |
| Exposed to domestic/interpersonal violence | 29.8% | 33.3% |
| Lost contact with close individual | 28.1% | 24.6% |
| Family/Close individual in jail or prison | 28.1% | 28.1% |
| Forced to leave home | 26.3% | 17.5% |
| Serious, accidental fall | 24.6% | 22.8% |
| Seriously sick/diagnosed with chronic illness | 24.6% | 31.6% |
| New caregiver moved into home | 22.8% | 29.8% |
| Car accident | 21.1% | 19.3% |
| Admitted to a hospital | 21.1% | 24.6% |
| Pet died | 19.3% | 17.5% |
| Caregiver alcoholism or drug use | 19.3% | 21.1% |
| Seriously sick/diagnosed with acute illness | 19.3% | 28.1% |
| Saw something terrible (e.g., fighting, robbery) | 15.8% | 17.5% |
| Close individual attempted or completed suicide | 15.8% | 12.3% |
| Attended childcare in unsafe location | 14.0% | 19.3% |
| Community violence | 14.0% | 10.5% |
| Caregiver died | 8.8% | 8.8% |
| Saw another person hurt/injured | 8.8% | 1.8% |
| Discrimination | 8.8% | 8.8% |
| Caregivers divorced | 7.0% | 7.0% |
| Broke a bone | 7.0% | 10.5% |
| Consistency ignored by peers | 7.0% | 0.0% |
| Poisoned | 5.3% | 5.3% |
| Physically attacked by another child | 5.3% | 5.3% |
| Natural disaster | 3.5% | 3.5% |
| Hit by a car | 1.8% | 0.0% |
| Seriously burned | 1.8% | 5.3% |
| Almost drowned | 1.8% | 3.5% |
| Sibling died | 1.8% | 0.0% |
| Peer/Friend died | 1.8% | 5.3% |
| Been in a fire | 1.8% | 3.5% |
| Removed from home due to neglect | 1.8% | 8.8% |
| Attacked by an animal | 0.0% | 3.5% |
| Involved in war/terrorism event | 0.0% | 0.0% |
| Mugged or robbed | 0.0% | 1.8% |
| Removed from home due to physical abuse | 0.0% | 1.8% |
| Kidnapped or taken hostage | 0.0% | 0.0% |
| Removed from home due to sexual abuse | 0.0% | 0.0% |
| Removed from home due to emotional abuse | 0.0% | 0.0% |
| Moved to new country | 0.0% | 0.0% |
| *N* = 57. Events ordered from highest to lowest percent endorsement on the questionnaire format | | |
